# Pyogenic Liver Abscess Correlates With Increased Risk of Acute Pancreatitis: A Population-Based Cohort Study

**DOI:** 10.2188/jea.JE20140152

**Published:** 2015-03-05

**Authors:** Shih-Wei Lai, Kuan-Fu Liao, Cheng-Li Lin, Pei-Chun Chen

**Affiliations:** 1 College of Medicine, China Medical University, Taichung, Taiwan; 2 Department of Family Medicine, China Medical University Hospital, Taichung, Taiwan; 3 Graduate Institute of Integrated Medicine, China Medical University, Taichung, Taiwan; 4 Department of Internal Medicine, Taichung Tzu Chi General Hospital, Taichung, Taiwan; 5 Management Office for Health Data, China Medical University Hospital, Taichung, Taiwan; 6 Clinical Informatics & Medical Statistics Research Center, Chang Gung University, Tao-Yuan, Taiwan; 7 Research Services Center for Health Information, Chang Gung University, Tao-Yuan, Taiwan

**Keywords:** acute pancreatitis, alcoholism, biliary stone, diabetes mellitus, pyogenic liver abscess

## Abstract

**Background:**

The aim of this study was to explore whether there is a relationship between pyogenic liver abscess (PLA) and subsequent risk of acute pancreatitis in Taiwan.

**Methods:**

Using inpatients claims data from the Taiwan National Health Insurance Program, we identified 30 866 subjects aged 20–84 years with the first-attack of PLA from 2000 to 2010 as the PLA group and randomly selected 123 464 subjects without PLA as the non-PLA group. The incidence of the first attack of acute pancreatitis at the end of 2010 and the risk associated with PLA and other comorbidities were measured.

**Results:**

The overall incidence of acute pancreatitis was 3.84-fold greater in the PLA group than in the non-PLA group (4.61 vs 1.19 events per 1000 person-years; 95% CI, 3.43–4.29). After controlling for potential confounding factors, the adjusted hazard ratio of acute pancreatitis was 3.00 (95% CI, 2.62–3.43) for the PLA group, as compared to the non-PLA group. Further analysis showed that compared to subjects with neither PLA nor comorbidities, patients with PLA and hypertriglyceridemia, biliary stones, alcoholism, or hepatitis C had greater risk of acute pancreatitis than those with PLA alone.

**Conclusions:**

PLA correlates with increased risk of subsequent acute pancreatitis. Comorbidities, including hypertriglyceridemia, biliary stones, alcoholism, and hepatitis C, may enhance the risk of developing acute pancreatitis.

## INTRODUCTION

Pyogenic liver abscess (PLA) is a global public health problem associated with severe morbidity and mortality. In Jepsen et al’s study in Denmark, the overall incidence of PLA was 10.7/1 000 000 person-years from 1977 to 2002.^1^ The annual incidence of PLA increased from 2.7/100 000 population in 1994 to 4.1/100 000 population in 2005 in the US^2^ and from 11.15/100 000 population in 1996 to 17.59/100 000 population in 2004 in Taiwan.^3^ The incidence varies worldwide depending on the populations studied, but incidence is much lower in western countries than in others. In addition to being life-threatening in the acute stage, severe consequences after the acute stage have been reported. Recent studies have illustrated that PLA is associated with increased risk of gastrointestinal cancers, including liver cancer, colorectal cancer, small intestine cancer, biliary tract cancer, and pancreatic cancer.^4^^–^^6^ Other studies have shown that patients with PLA during the first-year follow-up period are also at high risk of developing stroke^7^ and subsequent infections, including pneumonia, pulmonary abscess, pleural empyema, renal abscess, perinephric abscess, epidural spinal abscess, splenic abscess, prostatic abscess, and endophthalmitis^8^^–^^11^; however, acute pancreatitis has not been mentioned until now.

Acute pancreatitis is also a public health problem associated with severe morbidity and mortality. The incidence of first-attack acute pancreatitis ranged from 4 to 45 per 100 000 per year between 1961 and 2009 worldwide,^12^^–^^14^ depending on the study site. The case fatality rate of acute pancreatitis has improved in recent years, but still ranges from 7.5% to 3.3%.^12^^–^^15^ Epidemiological studies have revealed that diabetes mellitus, biliary stones, alcoholism, hypertriglyceridemia, and viral hepatitis are associated with increased risk of acute pancreatitis,^12^^,^^13^^,^^16^^–^^18^ and some of these factors are also associated with PLA risk.^3^^,^^19^^,^^20^

Previous studies have documented that a hepatic inflammatory process arising from PLA or non-alcoholic fatty liver disease can partially explain the increased risk of subsequent stroke.^7^^,^^21^ Therefore, based on the above-mentioned epidemiological evidence and inflammation theory, we proposed a biologically plausible hypothesis linking PLA to acute pancreatitis because both conditions are involved in the inflammatory process of the hepatic-biliary-pancreatic system. If PLA substantially correlates with increased risk of developing acute pancreatitis, interventions could be performed to reduce the risk of acute pancreatitis for PLA patients, such as controlling other comorbidities. Knowledge of the association between PLA and acute pancreatitis would be of help in the management of PLA patients. Therefore, we conducted a retrospective cohort study to explore whether PLA patients are at increased risk of developing acute pancreatitis and whether the risk associated with PLA is enhanced by other comorbidities.

## MATERIALS AND METHODS

### Data sources

This retrospective cohort study was based on data from the reimbursement claims of the universal National Health Insurance Program, which was implemented in Taiwan in March 1995 and provides health coverage to 99% of the 23 million residents living in Taiwan.^22^ National Health Research Institutes in Taiwan established a National Health Insurance Research Database containing multiple datasets, which includes registry for beneficiaries and information on medical claims. To protect personal privacy, the National Health Insurance Research Database was decoded with patient identifications scrambled before its release to public access for research. In the database, the diagnostic codes are in the format of the International Classification of Diseases, Ninth Revision, Clinical Modification (ICD-9-CM). The diagnostic accuracy of major diseases, such as PLA, acute pancreatitis, or diabetes mellitus, has been validated in previous studies.^6^^,^^18^^,^^23^ The study was approved by the Institutional Review Board of China Medical University and Hospital in Taiwan (CMU-REC-101-012).

### Participants

**Figure 1.**
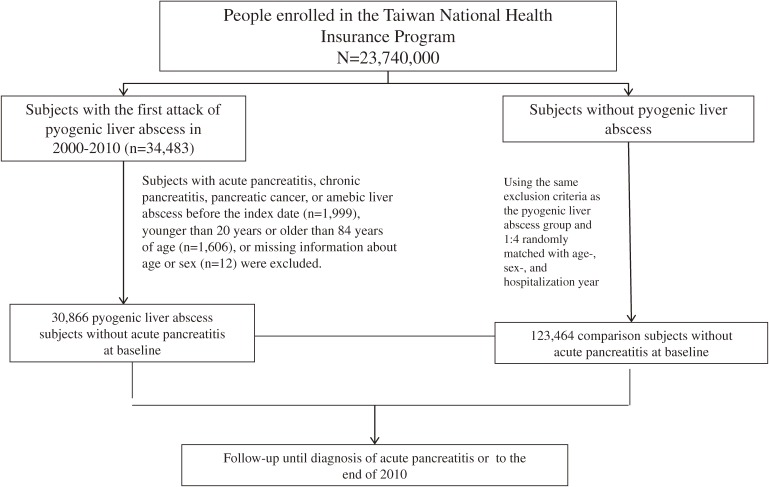
Flow chart of selection of study subjects.

Figure 1 shows the selection process of study subjects. We used inpatient claims to identify subjects with first-attack PLA (ICD-9-CM 572.0) during the period of 2000–2010 as the PLA group. The date of discharge with a diagnosis of PLA was defined as the index date. Four folds of comparison subjects without PLA were randomly selected from the National Health Insurance Research Database as the non-PLA group. The non-PLA subjects were matched with the PLA subjects by sex, age (every 5-year span), and year of hospitalization. In both groups, we excluded subjects diagnosed with acute pancreatitis, chronic pancreatitis, pancreatic cancer, and amebic liver abscess before the index date. Subjects less than 20 years old or more than 84 years old and those without information on sex or age were also excluded (Figure 1).

### Outcome measurement

All study subjects were followed until they were diagnosed with the first attack of acute pancreatitis, which was identified based on the hospitalization records during the follow-up period. The follow-up period was from the index date to the date of acute pancreatitis diagnosis, withdrawal from National Health Insurance Program, or the end of 2010.

### Comorbidity assessment

Risk factors for acute pancreatitis were identified in all subjects. Diabetes mellitus, hypertriglyceridemia, biliary stones, alcoholism, and hepatitis C were identified according to diagnoses in the hospitalization record data prior to the index date. All disorders were diagnosed using ICD-9-CM (eTable).

### Statistical analysis

We used χ^2^ tests to compare descriptive parameters for demographic status and baseline comorbidities between subjects with and without PLA. Continuous variables were tested with *t*-tests to compare differences between subjects with and without PLA. We used the Kaplan-Meier method to estimate cumulative incidences of acute pancreatitis for the PLA group and the non-PLA group, and we used the log-rank test to compare the difference between the two groups. The hazard ratios (HRs) and 95% confidence intervals (CIs) of acute pancreatitis risk were calculated for the PLA group compared to the non-PLA group using a Cox proportion hazard regression model. The multivariable Cox models were simultaneously adjusted for demographic characteristics and comorbidities. The interaction effects between comorbidity and PLA were also assessed in the Cox models. We computed the HRs for periods of follow-up of <3 months, <6 months, and 1–5 years, to observe whether the strength of associations differs over time. The proportional hazard assumption was assessed by including the interaction term of the predictors and time in the model. All analyses were performed using SAS version 9.2 (SAS institute Inc., Cary, NC, USA), with the significance level set to 0.05 on a two-tailed test.

## RESULTS

### Baseline characteristics of the study population

We identified 30 866 subjects in the PLA group and 123 464 subjects in the non-PLA group with similar distributions in sex and age (Table 1). The mean age of subjects in both groups was 60 years. Of PLA subjects, half were 40–64 years of age and only 9% were younger than 40 years. Male subjects accounted for 62.4% of PLA and non-PLA subjects. The mean (median) follow-up periods were 3.86 (3.21) years in the PLA group and 4.81 (4.54) years in the non-PLA group. Diabetes mellitus, hypertriglyceridemia, biliary stones, alcoholism, and hepatitis C were more prevalent in the PLA group (*P* < 0.0001 for all).

**Table 1.** Baseline characteristics between pyogenic liver abscess group and non-pyogenic liver abscess group

Characteristic	Pyogenic liver abscess				*P* value

	No *n* = 123 464		Yes *n* = 30 866		
	
	*n*	(%)	*n*	(%)	
Sex					0.99
Male	77 016	62.4	19 254	62.4	
Female	46 448	37.6	11 612	37.6	
Age group (years)					0.99
20–39	11 125	9.0	2778	9.0	
40–64	61 887	50.1	15 475	50.1	
65–84	50 452	40.9	12 613	40.9	
Age (years), mean (SD)^a^	60.0	14.3	60.2	14.1	<0.0001
Follow-up time (years), mean^a^	4.81	4.54	3.86	3.21	<0.0001
Baseline comorbidities					
Diabetes mellitus	8843	7.16	12 592	40.79	<0.0001
Hypertriglyceridemia	588	0.48	353	1.14	<0.0001
Biliary stones	2852	2.31	6261	20.28	<0.0001
Alcoholism	390	0.32	336	1.09	<0.0001
Hepatitis C	779	0.63	1161	3.76	<0.0001

SD, standard deviation.

Data are presented as the number of subjects in each group, with percentages given in parentheses or mean, with median given in parentheses.

Chi-square test and ^a^*t*-test comparing subjects with and without pyogenic liver abscess.

### Risk of acute pancreatitis associated with PLA

**Figure 2.**
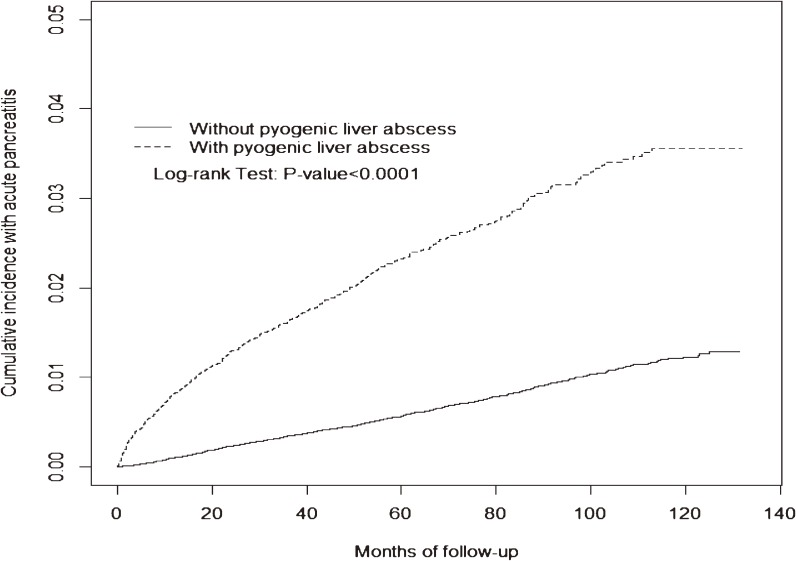
The Kaplan-Meier cumulative incidence of acute pancreatitis in the pyogenic liver abscess group and the non-pyogenic liver abscess group (3.56% vs 1.23% at the end of follow-up; *P* < 0.0001).

Figure 2 shows that the cumulative incidence of acute pancreatitis was higher in the PLA group than that in the non-PLA group (3.56% vs 1.23%; *P* < 0.0001) at the end of follow-up. The overall incidence of acute pancreatitis was 4.61 per 1000 person-years in the PLA group and 1.19 per 1000 person-years in the non-PLA group (crude HR 3.84; 95% CI, 3.43–4.29) (Table 2). After controlling for comorbid diabetes mellitus, hypertriglyceridemia, gallstones, alcoholism, and hepatitis C, the adjusted HR of acute pancreatitis for the PLA group compared to the non-PLA group was 3.00 (95% CI, 2.62–3.43). The risk fell over time but persisted through the follow-up period (Table 2). The highest risk occurred during the first 3 months of follow-up (adjusted HR 14.7; 95% CI, 8.82–24.6) and the adjusted HR decreased, to 2.31 at 3 years of follow-up (95% CI, 1.57–3.41). The increased risk in the PLA group remained for the 5 years of follow-up. In the analysis on the entire follow-up period, the proportional hazard assumption was not met. These observations consistently suggested a time-varying association between PLA and acute pancreatitis, particularly in the first 3 months of follow-up.

**Table 2.** Incidence density and hazard ratios of acute pancreatitis associated with pyogenic liver abscess

	Non-pyogenic liver abscess				Pyogenic liver abscess				Crude HR (95% CI)	Adjusted HR^b^ (95% CI)
	
	*n*	Event	Person- years	Rate^a^	*n*	Event	Person- years	Rate^a^		
All	123 464	705	593 588	1.19	30 866	550	119 242	4.61	3.84 (3.43, 4.29)	3.00 (2.62, 3.43)
Follow-up period										
<3 months	123 464	21	30 423	0.69	30 866	96	7172	13.4	19.3 (12.0, 30.9)	14.7 (8.82, 24.6)
<6 months	119 887	48	59 953	0.80	27 269	141	13 757	10.3	7.48 (4.64, 12.0)	5.25 (3.02, 9.13)
<1 year	116 253	122	116 278	1.05	25 549	219	25 887	8.46	4.90 (3.56, 6.73)	3.87 (2.67, 5.62)
<2 years	109 021	253	217 908	1.16	23 031	319	46 943	6.80	3.69 (2.84, 4.79)	2.89 (2.12, 3.93)
<3 years	94 400	350	305 569	1.15	19 179	377	64 583	5.84	2.97 (2.15, 4.12)	2.31 (1.57, 3.41)
<4 years	80 990	426	380 125	1.12	16 091	429	79 219	5.42	3.49 (2.45, 4.96)	2.89 (1.89, 4.40)
<5 years	68 286	502	442 197	1.14	13 252	474	91 157	5.20	3.08 (2.13, 4.45)	2.15 (1.37, 3.36)

CI, confidence interval; HR, hazard ratio.

^a^Incidence rate: per 1000 person-years.

^b^Adjusted for age, diabetes mellitus, hypertriglyceridemia, biliary stone, alcoholism and hepatitis C.

### Interaction effects of PLA and comorbidities on risk of acute pancreatitis

Table 3 shows the interaction effects of PLA and comorbidities on risk of acute pancreatitis. When compared to subjects without PLA and no comorbidities, the adjusted HR was 3.62 (95% CI, 3.07–4.28) for subjects with PLA alone and 2.39 (95% CI, 1.99–2.88) for those with at least one comorbidity but without PLA. The HR increased to 5.09 (95% CI, 4.46–5.81) for subjects with PLA and at least one comorbidity. Co-existing with hypertriglyceridemia, (adjusted HR 5.31; 95% CI, 2.65–10.7), biliary stones (adjusted HR 6.65; 95% CI, 5.65–7.81), alcoholism (adjusted HR 7.92; 95% CI, 3.94–15.9), and hepatitis C (adjusted HR 6.47; 95% CI, 4.23–9.90) enhanced the risk of acute pancreatitis in subjects with PLA. Compared to subjects without PLA and diabetes mellitus, the HR in subjects with diabetes mellitus alone was 1.75 (95% CI, 1.39–2.21). The HR increased to 4.32 (95% CI, 3.78–4.94) in subjects with PLA alone, and remained at a similar level in subjects with both PLA and diabetes mellitus (HR 3.94; 95% CI, 3.38–4.60). The results indicated that diabetes mellitus was associated with increased risk of acute pancreatitis in the non-PLA group but not in the PLA group.

**Table 3.** Cox proportional hazards regression analysis for interaction effects of pyogenic liver abscess and comorbidity on risk of acute pancreatitis

Variable		Person-years	Event	Adjusted HR^a^ (95% CI)
Pyogenic liver abscess	Comorbidity^b^			
No	No	548 045	562	1 (Reference)
No	Yes	45 543	143	2.39 (1.99, 2.88)
Yes	No	53 807	188	3.62 (3.07, 4.28)
Yes	Yes	65 435	362	5.09 (4.46, 5.81)

Pyogenic liver abscess	Diabetes mellitus			
No	No	560 767	621	1 (Reference)
No	Yes	32 821	84	1.75 (1.39, 2.21)
Yes	No	71 188	331	4.32 (3.78, 4.94)
Yes	Yes	48 053	219	3.94 (3.38, 4.60)

Pyogenic liver abscess	Hypertriglyceridemia			
No	No	591 209	699	1 (Reference)
No	Yes	2380	6	1.77 (0.79, 3.96)
Yes	No	117 984	542	3.95 (3.53, 4.42)
Yes	Yes	1259	8	5.31 (2.65, 10.66)

Pyogenic liver abscess	Biliary stones			
No	No	582 496	654	1 (Reference)
No	Yes	11 092	51	3.19 (2.40, 4.25)
Yes	No	96 325	359	3.46 (3.04, 3.93)
Yes	Yes	22 918	191	6.65 (5.65, 7.81)

Pyogenic liver abscess	Alcoholism			
No	No	592 198	697	1 (Reference)
No	Yes	1391	8	5.08 (2.53, 10.22)
Yes	No	118 149	542	3.96 (3.54, 4.43)
Yes	Yes	1094	8	7.92 (3.94, 15.93)

Pyogenic liver abscess	Hepatitis C			
No	No	591 159	691	1 (Reference)
No	Yes	2430	14	4.09 (2.41, 6.95)
Yes	No	116 667	528	3.95 (3.53, 4.42)
Yes	Yes	2576	22	6.47 (4.23, 9.90)

CI, confidence interval; HR, hazard ratio.

^a^Adjusted HR: Adjusted for age and sex.

^b^Patients with any one of the comorbidities (diabetes mellitus, hypertriglyceridemia, biliary stone, alcoholism and hepatitis C) were classified as the comorbidity group.

## DISCUSSION

Overall, this is the first population-based cohort study that attempts to explore the risk of subsequent acute pancreatitis in PLA patients and the effects associated with comorbidities in Taiwan. No relevant study has previously reported this association. We used a well-preserved database in Taiwan that has been analyzed to explore numerous epidemiological problems with considerable contribution to the literature. The accuracy and reliability of the diagnosis codes in the present study have been validated in high-quality journals.^6^^,^^18^

In the present study, the incidence of acute pancreatitis in the PLA group was approximately 3.8-fold greater than in the non-PLA group. The incidence among PLA patients in this study was even moderately higher than that in diabetic patients reported by Shen et al in Taiwan (4.61 vs 2.98 per 1000 person-years).^24^ The HR of acute pancreatitis was higher in the PLA-only group than that in the diabetes-only group (4.32 vs 1.75; Table 3). No additional effect was observed in patients with both PLA and diabetes mellitus. This suggests that PLA has a much greater contribution to risk of developing acute pancreatitis than diabetes mellitus.

**Figure 3.**
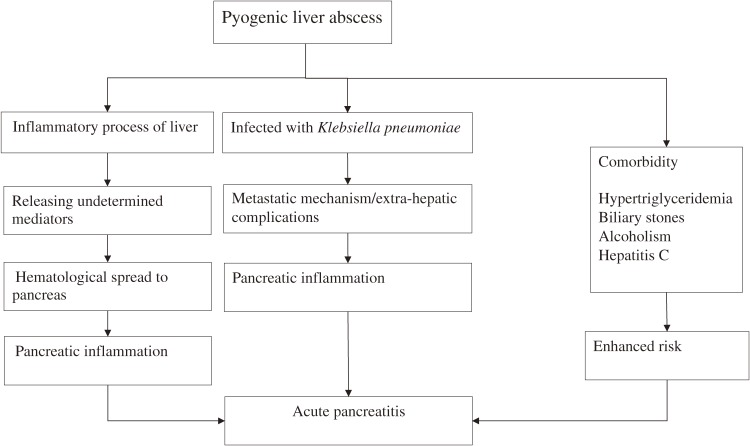
Potential mechanisms of acute pancreatitis following pyogenic liver abscess.

While the cause of the association between PLA and acute pancreatitis cannot be clarified in this observational study, we reviewed the relevant studies to explain the mechanism between PLA and risk of acute pancreatitis (Figure 3). First, because hepatic inflammatory processes due to from PLA or non-alcoholic fatty liver disease can increase the risk of subsequent stroke,^7^^,^^21^ we think some undetermined mediators related to the hepatic inflammatory process of PLA may exacerbate pancreatic inflammation via hematological spread. We also noticed that, even without any comorbidity, patients with PLA alone still had a 3.62-fold increased risk of acute pancreatitis (Table 3). This finding suggests that PLA has a unique and substantial effect on acute pancreatitis risk.

Second, another possible mechanism of the inflammation theory could involve extra-hepatic complications of PLA through a metastatic mechanism. In Taiwan, 73%–87.9% of all PLA cases were caused by *Klebsiella pneumoniae* alone, and the others could be caused by mixed flora.^10^^,^^25^ Previous studies reported that PLA patients infected with *Klebsiella pneumoniae* were more likely to have extra-hepatic complications and metastasis to the whole body than those with other types of PLA.^10^^,^^26^^,^^27^ These extra-hepatic complications and metastasis may exacerbate pancreatic inflammation.

Finally, we found that the increased risk of acute pancreatitis persisted over long periods, but risk was particularly increased during the first 3 months. This finding may at least partially support an inflammatory process progressing from PLA to subsequent acute pancreatitis through a metastatic mechanism. We suggest that clinicians should be particularly cautious of acute pancreatitis risk after diagnosing PLA, particularly during the first 3 months after PLA diagnosis.

Epidemiological studies have shown that acute pancreatitis is consistently associated with other comorbidities.^12^^,^^13^^,^^16^^–^^18^ The present study revealed that PLA alone had an HR of 3.62 in association with developing acute pancreatitis. The HR increased to 5.31 in PLA patients with hypertriglyceridemia, 6.47 in PLA patients with hepatitis C, 6.65 in PLA patients with biliary stone, and 7.92 in PLA patients with alcoholism (Table 3). This indicates that these comorbidities enhance the risk of developing acute pancreatitis in patients with PLA. Even after treatment of PLA, risk may remain high, which could partially explain why the probability of developing acute pancreatitis persisted throughout the follow-up period (Table 2). Whether improving these comorbidities in PLA patients can reduce the risk of developing acute pancreatitis needs further investigation.

Limitations of the present study should be addressed. First, due to inherent limitations of the insurance database, we were unable to determine the causative mechanism of PLA in this study. Thus, we could not explore whether *Klebsiella pneumoniae* or other organisms influenced risk of acute pancreatitis. Nevertheless, our findings suggest that the influence of bacterial infection in PLA and acute pancreatitis deserves attention in future research. Second, also due to limitations in the database, there is no information concerning the severity of PLA or acute pancreatitis. Thus, we were unable to explore whether different severities of PLA have different impacts on risk of acute pancreatitis. Third, due to the same limitations, the exact etiologies of PLA and acute pancreatitis of the included patients were not recorded. According to literature review, PLA and acute pancreatitis may share some risk factors, such as alcoholism, biliary stones, and diabetes mellitus.^3^^,^^18^^–^^20^^,^^27^ Fourth, although the literature shows that acute pancreatitis can be induced by iatrogenic endoscopic retrograde cholangiopancreatography (ERCP),^28^ we could not assess whether the included patients were admitted because of biliary-related symptoms due to therapeutic ERCP or whether they were admitted due to other causes that may have been complicated by iatrogenic ERCP. That is, we were unable to determine whether ERCP is a cause or an effect for acute pancreatitis, so we did not include ERCP in our analysis. However, future research on the association between ERCP and acute pancreatitis is warranted. Fifth, we were unable to clarify whether or not the effects of PLA on the development of acute pancreatitis are specific to PLA, due to the observational nature of the present study. The potential involvement of other inflammatory processes, like pneumonia or pyelonephritis, on increasing the risk of acute pancreatitis needs further exploration. Sixth, congenital abnormalities of the pancreaticobiliary system, such as pancreaticobiliary maljunction and pancreas divisum, may be associated with acute pancreatitis.^29^ We attempted to search the ICD-9-CM, but no specific codes for these diseases could be found. Therefore, we could not include these diseases in our analysis. Last, we used the inpatient database to identify all diseases. The impact of excluding outpatient data would be minimal to PLA and acute pancreatitis because the two conditions are likely to lead to hospitalization. However, some comorbidities such as diabetes mellitus, which would less likely to lead hospital admission, may not be recorded in inpatient claims if patients only received treatment in outpatient clinics. Among diabetes patients in the PLA group, who had at least 1 hospitalization for PLA treatment, the comorbid diabetes might be recorded during PLA hospitalizations and could be identified. However, among diabetes patients in the non-PLA group who received anti-diabetes treatment in outpatient clinics and never had hospitalization, their diabetes would not be identified. This may partially explain the much lower prevalence of comorbidities in the non-PLA group. Misclassification of comorbidities could reduce the ability to control for confounding. However, it is unlikely that residual confounding would entirely explain the association, especially with the very large effect size observed in the early years of follow-up.

Despite the above limitations, the present study has numerous advantages. It was based on a nationwide population-based database with a relatively large sample size and with high statistical power. This advantage increases the accuracy of identification of the studied diseases (such as PLA and acute pancreatitis). Therefore, the increased risk associated with PLA and other comorbidities could be discerned. In addition, since patients with PLA were managed in the hospital setting, patients were discharged from the hospital only after successful treatment. At present, no clinical guideline suggests that outpatients with PLA should be surveyed more frequently to increase detection rates of complications, as compared to other historical controls. Therefore, selection bias based on using an inpatient sample is unlikely to occur.

We conclude that patients with PLA are at higher risk of developing acute pancreatitis. The risk associated with PLA is enhanced by comorbidities, including hypertriglyceridemia, biliary stones, alcoholism, and hepatitis C. Future studies are needed to clarify the biological mechanism by which PLA is associated with risk of developing acute pancreatitis and to explore which causative organism of PLA mainly influences the risk.

## ONLINE ONLY MATERIAL

eTable. Disorders in the study.
