# Supplementary material for: Pyogenic Liver Abscess Correlates With Increased Risk of Acute Pancreatitis: A Population-Based Cohort Study
Source: J Epidemiol. 2015 Mar 5;25(3):246–53. doi: 10.2188/jea.JE20140152 (PMC4341002; doi:10.2188/jea.JE20140152)
Supplement: eTable. [file je-25-246-s001.pdf]

eTable. Disorders in the study

**Disorders**	**ICD-9-CM code**
Pyogenic liver abscess	572.0
Acute pancreatitis	577.0
Chronic pancreatitis	577.1
Pancreatic cancer	157
Amebic liver abscess	006.3
Diabetes mellitus	250
Hypertriglyceridemia	272.1
Biliary stones	574
Alcoholism	291, 303, 305.00, 305.01, 305.02, 305.03, 790.3 and V11.3
Hepatitis C	V02.62, 070.41, 070.44, 070.51 and 070.54
